# Synergistic Impaired Effect between Smoking and Manganese Dust Exposure on Pulmonary Ventilation Function in Guangxi Manganese-Exposed Workers Healthy Cohort (GXMEWHC)

**DOI:** 10.1371/journal.pone.0116558

**Published:** 2015-02-09

**Authors:** Fenfen Wang, Yunfeng Zou, Yuefei Shen, Yaoqiu Zhong, Yingnan Lv, Damin Huang, Kangcheng Chen, Qin Li, Li Qing, Bing Xia, Cheng Su, Shuyan Ma, Xiaobo Yang

**Affiliations:** 1 Department of occupational health and environmental health, School of Public Health, Guangxi Medical University, Nanning, Guangxi, China; 2 Department of Toxicology, School of Public Health, Guangxi Medical University, Nanning, Guangxi, China; 3 Department of Neurology, The First Affiliated Hospital of Guangxi Medical University, Nanning, Guangxi, China; 4 Center for Genomic and Personalized Medicine, Guangxi Medical University, Nanning, Guangxi, China; MOE Key Laboratory of Environment and Health, School of Public Health, Tongji Medical College, Huazhong University of Science and Technology, CHINA

## Abstract

**Purpose:**

The aims of this study were to investigate the effects of manganese (Mn) dust exposure on lung functions and evaluate the potential synergistic effect between smoking and Mn dust exposure among refinery workers.

**Methods:**

A retrospective study including 1658 workers in a ferromanganese refinery was conducted, with subjects who were from the Guangxi manganese-exposed workers healthy cohort (GXMEWHC). Based on the Mn manganese cumulative exposure index (Mn-CEI), all subjects were divided into the low exposure group (n = 682) and the high exposure group (n = 976). A pulmonary function test was performed using an electronic spirometer, including the values and percentages of FVC, FEV_1_, FEV_1_/FVC, MMEF, PEFR, MVV, respectively.

**Results:**

No significant effect of Mn dust exposure on the pulmonary function was found in the female workers (all *p*>0.05). However, there was an obvious decrease in the male workers in the high exposure group compared with those in the low exposure group (FVC -60 ml, FEV_1_ -120 ml, MMEF -260 ml/s, MVV -5.06 L, all *p*<0.05). In the high exposure group, the reduction in FVC% predicted, MMEF and MMEF% predicted was 1.0%, 210 mL/s, and 4.9%, respectively. In particular, among the exposed subjects smokers had a statistically significant decrease in lung function compared with non-smokers and the reduction in FVC% predicted, MMEF and MMEF% predicted was 1.0%, 210 mL/s, and 4.9%, respectively (*p*<0.05). Partial correlation analysis showed that there was also negative correlation between Mn-CEI and decreased changes in MMEF (r = -0.159, *p* = 0.018) and also MMEF% predicted (r = -0.163, *p* = 0.015).

**Conclusions:**

Mn dust can impair the pulmonary ventilation function of male workers but not females, and individual smoking habits and manganese exposure had a synergistic effect on the lung function decrease.

## Introduction

Manganese (Mn) is an essential nutrient and it is necessary to maintain human health. In addition to food intake, environmental exposure is also the way to absorb Mn. The combustion products of methylcyclopentadienyl manganese tricarbonyl (MMT) in unleaded gasoline has been discharged approximately 30% Mn phosphate/sulfate mixture into the environment[1]. Similarly, a large number of occupational workers who work in industries such as welding, smelting, mining, ferroalloy and dry battery production are also exposed to high concentrations of Mn dust [2,3].

The major routes of Mn entry and absorption are the respiratory and gastrointestinal tracts[4], of which the proportion is 60% to 70% and 1% to 5% respectively [5].Therefore, the lung is one of the main target organs affected by long-term exposure to Mn dust [6]. Dorman et al. [7] reported that high-dose subchronic Mn sulfate inhalation was associated with increasing lung Mn concentrations. Previous studies also showed that long-term exposure to Mn dust led to workers’ lung function decrease in FVC, FEV_1_ and FEV_1_% pred [8,9]. However, the conclusions of these studies were inconsistent, and most of them were conducted without considering the long-term cumulative effect between manganese cumulative exposure index (Mn-CEI) and respiratory function impairment. Additionally, these included subjects were mostly male workers but not female workers. Therefore, based on our constructed Guangxi manganese-exposed workers healthy cohort (GXMEWHC) [10], we can further explore the effects of Mn dust exposure on the respiratory health in the cohort population.

Previous studies have shown that smoking is one of the most important identified causes of chronic obstructive pulmonary disease (COPD) and an aetiological factor for pancreatic cancer [11], and cigarette smoking is a key lifestyle factor that affects respiratory health [12,13]. In addition, smoking is considered to have a synergistic effect with tile dust exposure [14]. Langhammer et al. [15] found that smoking was associated with reduced lung function and increased prevalence of respiratory symptoms. In the GXMEWHC population, about 50% of male workers have the habit of smoking, but few studies to date have shown the potential interaction between smoking and Mn dust exposure.

Thus, the objectives of this study were undertaken to further substantiate early changes in the respiratory system in the GXMEWHC with long-term exposure to Mn dust and to evaluate the potential synergistic effect between Mn dust and smoking.

## Materials and Methods

### Study population

We established the GXMEWHC which consists of 1888 workers in the ferromanganese refinery from July 2011 to September 2012, as previously described [16], 1658 workers were finally recruited according to inclusion criteria. The main criteria for participation were employment for at least 3 months, no diabetes mellitus or serious kidney or liver diseases, lung diseases that are probably related to Mn exposure, and no physical disability or surgical history. Additionally, the exclusion criteria included chronic obstructive pulmonary diseases and unwillingness to participate in the interview.

The study was approved by the ethics and human subjects committee of Guangxi Medical University. All participants signed informed consent forms before the start of this study.

### Questionnaire

Basic demographic information was collected using a specifically designed questionnaire after obtaining written informed consent. The questionnaire also included drinking status, smoking status, disease history, medication and occupational history, and socio-economic factors. Drinking status was defined as current drinking (drinking at least once each week for more than 3 months), former drinking (the person had stopped drinking for at least 3 months) and never drinking. Smoking status was defined as current smoking (smoking at least one cigarette daily for more than 3 months), former smoking (stopped smoking for at least 3 months) and never smoking. We calculated the smoking index from the number of cigarettes smoked per day and multiplied it by years of smoking.

### Pulmonary function tests

The pulmonary function was recorded by a spirometer (SiChuan sikeda S-980A Technology Co., Ltd. China) according to the standards of the American Thoracic Society[17]. We measured the values and percentages of forced vital capacity (FVC), forced expiratory volume at one second (FEV_1_), the ratio of forced expiratory volume at one second (FEV_1_%), maximal med-expiratory flow curve (MMEF), peak expiratory flow ratio (PEFR), and maximal voluntary ventilation (MVV).

FEV_1_ (L) and FVC (L) were measured with the best value of three forced expiratory manoeuvres in the standing position. The largest and second-largest FVC measurements should be within 100 ml or 0.5% of each other when expressed as a percentage of the larger FVC. The FVC and FEV_1_ values in per cent of predicted (% pred) were calculated according to the ECCS reference values [18]. The mean per cent predicted value was based on subject age, height, weight, sex and ethnic group as calculated and adjusted by the spirometer device. The percentage predicted lung values were observed capacities as measured by a spirometer divided by predicted or expected capacities multiple by 100 [19].

%predicted lung value=(observed capacities/expected capacities)*100

All respiratory function tests were carried out at a fixed time of the day (8.00 am to 12.00 pm) to minimize the diurnal variation [20].

### Manganese exposure assessment

We had recorded the technological processes of production and the situation of potential Mn pollution in this company. We detected the individual levels of Mn by individual samplers during on-job time. Air samples were collected by FC-2 dust samplers according to the standard specification issued by the Ministry of Health in China-Specifications of air sampling for hazardous substances monitoring in the workplace (GBZ 159–2004), and the method had been described in detail in previous studies [21]. Permissible concentration-time-weighted average (PC-TWA) is the average permissible exposure levels on the regulation eight hours working day weighting by time. The cumulative exposure index (CEI) is calculated which combines PC-TWA and the working seniority. As Mn external exposure index, the Mn-CEI was calculated for each subject referred to the individual airborne monitoring in the working time and also breaking time [10].

We divided all subjects into two groups based on the Mn-CEI [22] including the low exposure group (Mn-CEI<1.00 mg/m^3^.year) and the high exposure group (Mn-CEI≥1.00 mg/m^3^.year).

### Statistical analysis

All analyses were performed by the SPSS statistical program, version 16.0. Analysis of covariance was used to compare continuous variables between groups by estimating the mean ± SE. The discrete variables were evaluated using a Chi-Square test. We used analysis of covariance (ANOVA) to compare the differences of lung functions between the high exposure group and the low exposure group, adjusted by the level of age, height, weight and smoking index. Partial correlation analysis was applied for the relation between Mn-CEI and pulmonary function values of workers, adjusted by the above confounding factors. All statistical tests were two-tailed and the level of significance was achieved at *p* < 0.05.

## Results

### Manganese concentration in the workplaces

The high exposure workplaces mainly included smelters, human crushing worker and craneman, and the Mn concentration in these workplaces ranged from 0.015 to 0.363 mg/m^3^. Additionally, the low exposure workplaces were mainly comprised auxiliary workers, such as car driver, alkali recovery worker and electrician, and the Mn concentrations in these workplaces ranged from 0.004 to 0.140 mg/m^3^. The Mn-CEI was in the range 0.010–10.300 mg/m^3^.year and median was 1.290 mg/m^3^. year in all workers. The details of the PC-TWA and the Mn-CEI results are shown in S1 Table and S2 Table.

### Characteristics of the study population

As presented in Table 1, a total of 1658 subjects enter this study, including low exposure group (n = 682) and high exposure group (n = 976). The low exposure group comprised 407 men (59.7%) and 275 women (40.3%), and the high exposure group comprised 632 men (64.8%) and 345 women (35.3%). The average age was 37.6 years for the low exposure workers and 42.6 years for the high exposure workers. Of the low exposure workers, 348 (51.0%) were current smokers and 350 (51.3%) current drinkers. Of the high exposure workers, 289 (29.6%) were current smokers and 431 (44.1%) current drinkers. Significant difference was found between the low exposure group and the high exposure group regarding the mean age, seniority, smoking distribution, drinking distribution and gender, *p*<0.05, respectively.

**Table 1:** Demographic data of the study population.

	Low exposure group	High exposure group	
	0>Mn-CEI<1 (N = 682)	Mn-CEI≥1 (N = 976)	
Variables	Mean ± SD	Mean ± SD	*P* value
Age(years)	37.58±8.40	42.57±6.35	0.000
Seniority(years)	11.46±9.62	18.55±8.48	0.000
Height(cm)	163.27±7.64	162.93±7.35	0.361
Weight(kg)	59.71±9.41	60.26±9.57	0.248
Sex			
Male	407(59.7)	631(64.7)	0.039
Female	275(40.3)	345(35.3)	
Smoking status			
Current smoker	348(51.0)	289(29.6)	0.000
Former smoker	70(10.3)	52(5.3)	
Never smoke	264(38.7)	635(65.1)	
Drinking status			
Current drinker	350(51.3)	431(44.1)	0.002
Former drinker	113(16.6)	151(15.5)	
Never drink	219(32.1)	394(40.4)	

SD, standard deviation

### Effects of the Mn-CEI on lung function outcomes among males

Table 2 describes the effects of different variables on lung function, adjusted by the levels of age, height, weight and smoking index. Average FEV_1_, FEV_1_% pred, FEV_1_/FVC, FEV_1_/FVC% pred, MMEF, MMEF% pred, MVV and MVV% pred was lower in the high exposure workers than those in the the low exposure group (all *p*<0.05). Compared with the low exposure group, the adjusted impaired effects were-60 ml on FVC, -120 ml on FEV_1_, -260 ml/s on MMEF and 5.06 L on MVV in the high exposure group. There was a statistically significant difference between the high exposure group and the low exposure group in FEV_1_, FEV_1_/FVC, MMEF and MVV (*p*<0.05).

**Table 2:** Adjusted effects on lung function of males by the levels of age, height, weight and smoking index.

	Low exposure group		High exposure group			
	0>Mn-CEI<1 (N = 407)		Mn-CEI≥1 (N = 631)		
Variables	Mean	SE	Mean	SE	*P* value
FVC, L	4.26	0.04	4.20	0.03	0.223
FVC% pred	110.15	0.91	108.44	0.72	0.150
FEV_1_, L	3.67	0.03	3.55	0.30	0.003
FEV1% pred	112.82	0.99	109.96	0.78	0.003
FEV_1_/FVC	86.52	0.50	85.01	0.39	0.021
FEV_1_/FVC% pred	112.54	0.66	110.86	0.52	0.051
MMEF,L/s	4.67	0.07	4.41	0.05	0.003
MMEF% pred	104.92	1.48	98.95	1.18	0.002
PEFR,L/min	8.20	0.13	8.17	0.10	0.873
PEFR% pred	90.85	1.42	88.80	1.12	0.272
MVV,L	146.78	1.27	141.72	1.01	0.003
MVV% pred	147.01	1.30	142.44	1.03	0.011

The results of the analysis of covariance are shown adjusted for age (y) and height (cm), weight (kg) and smoking index. SE, standard error.

### Effects of the Mn-CEI on lung function outcomes among females

As shown in Table 3, the effects of different variables on lung function are adjusted by the level of age, height and weight. Average FVC, FVC% pred, FEV_1_, FEV_1_% pred, FEV_1_/FVC, MMEF, MMEF% pred, MVV and MVV% pred was higher in the high exposure workers than the low exposure group except FEV_1_/FVC% pred, but there was no statistically significant difference between the high exposure group and the low exposure group, all *p* >0.05.

**Table 3:** Adjusted effects on lung function of female workers by the levels of age, height and weight.

	Low exposure group		High exposure group		
	0<Mn-CEI<1 (N = 275)		Mn-CEI≥1 (N = 345)		
Variables	Mean	SE	Mean	SE	*P* value
FVC, L	3.11	0.03	3.16	0.03	0.298
FVC% pred	110.84	1.11	113.62	0.99	0.073
FEV_1_, L	2.67	0.03	2.70	0.03	0.412
FEV_1_% pred	110.76	1.20	113.53	1.07	0.087
FEV_1_/FVC	86.15	0.56	86.25	0.49	0.893
FEV_1_/FVC% pred	105.98	0.68	105.91	0.61	0.946
MMEF,L/s	3.39	0.06	3.40	0.05	0.860
MMEF% pred	98.54	1.63	100.17	1.45	0.466
PEFR,L/min	5.43	0.10	5.48	0.09	0.695
PEFR% pred	82.53	1.55	84.64	1.38	0.322
MVV,L	106.98	1.16	108.14	1.03	0.467
MVV% pred	127.15	1.35	129.58	1.20	0.191

The results of the analysis of covariance are shown adjusted for age (y) and height (cm), weight (kg). SE, standard error.

### Synergism between the Mn dust and smoking

Table 4 shows the results of potential synergism between Mn cumulative exposure (Mn-CEI) and smoking among male subjects. Confounding factors were adjusted including age, height, and weight. Compared with non-smokers, smokers have lower lung function values in both the low exposure group and the high exposure group. The averages of all lung parameters of the high exposure group were lower than the low exposure group. In the high exposure group, we also found that the adverse effects of manganese dust exposure on lung function were larger in smokers than in non-smokers. The reduction in FVC% pred, MMEF and MMEF% pred was 1.0%, 210 mL/s, and 4.9%, respectively. A similar pattern was found for other lung function parameters, although the reductions in lung function parameters were not statistically significant (*p*>0.05).

**Table 4:** Pulmonary function values for males by smoking habit per study stage.

	Low exposure group						High exposure group					
	0<Mn-CEI<1 (N = 407)						Mn-CEI≥1 (N = 631)					
	non-smoking (n = 113)		Smoking (n = 294)				non-smoking (n = 405)		Smoking (n = 226)			
Variables	Mean	SE	Mean	SE		*P* value	Mean	SE	Mean		SE	*P* value
FVC, L	4.40	0.06	4.39	0.04		0.889	4.19	0.05	4.09		0.04	0.094
FVC% pred	11.08	1.60	111.01	0.99		0.969	109.74	1.19	106.79		0.89	0.047
FEV_1_, L	3.79	0.06	3.79	0.04		0.918	3.51	0.04	3.46		0.03	0.377
FEV_1_% pred	113.30	1.81	113.58	1.12		0.896	110.03	1.28	107.62		0.95	0.132
FEV_1_/FVC	86.23	0.87	86.97	0.54		0.475	85.20	0.49	84.2		0.66	0.225
FEV_1_/FVC% pred	110.60	1.16	111.21	0.72		0.657	112.39	0.65	111.01		0.87	0.204
MMEF,L/s	4.71	0.12	4.85	0.07		0.307	4.46	0.09	4.25		0.06	0.052
MMEF% pred	101.57	2.60	105.08	1.61		0.253	102.66	1.96	97.73		1.47	0.045
PEFR,L/min	8.49	0.25	8.40	0.15		0.751	8.22	0.16		7.97	0.12	0.218
PEFR% pred	92.67	2.67	91.66	1.65		0.746	90.13	1.81		87.07	1.34	0.175
MVV,L	151.34	2.36	151.74	1.46		0.886	140.32	1.62		138.32	1.21	0.325
MVV% pred	149.17	2.36	147.62	1.46		0.576	144.06	1.70		140.58	1.27	0.101

### Partial correlation analysis between Mn-CEI and pulmonary function values

As presented in Table 5, in high exposure group, there was significant negative correlation between cumulative exposure to manganese and changes in MMEF, MMEF% pred (*p*<0.05). A similar pattern was found for other lung function parameters, although the reductions in lung function parameters were not statistically significant, *p*>0.05.

**Table 5:** Partial correlation analysis between Mn-CEI and pulmonary function values of workers.

	Male				Female	
Variables	r_1_ (N = 226)	*P* value	r_2_ (N = 405)	*P* value	r_3_ (N = 618)	*P* value
FVC, L	-0.072	0.287	-0.056	0.260	0.029	0.468
FVC% pred	-0.073	0.280	-0.021	0.677	0.050	0.216
FEV_1_, L	-0.087	0.198	-0.092	0.064	0.066	0.104
FEV_1_% pred	-0.107	0.111	-0.031	0.534	0.071	0.079
FEV_1_/FVC	-0.027	0.686	-0.067	0.177	0.039	0.332
FEV_1_/FVC% pred	-0.042	0.533	-0.037	0.456	0.035	0.392
MMEF,L/s	-0.159	0.018	-0.055	0.268	0.042	0.293
MMEF% pred	-0.163	0.015	-0.021	0.674	0.051	0.205
PEFR,L/min	-0.058	0.392	-0.040	0.420	0.074	0.067
PEFR% pred	-0.086	0.200	-0.040	0.424	0.086	0.079
MVV,L	-0.087	0.197	-0.089	0.074	0.066	0.103
MVV% pred	-0.043	0.525	-0.032	0.524	0.070	0.615

r_1_, the partial correlation coefficients of smoking male workers in the high exposure group

r_2_, the partial correlation coefficients of non-smoking male workers in the high exposure group

r_3_, the partial correlation coefficients of female workers

**Figure 1:**
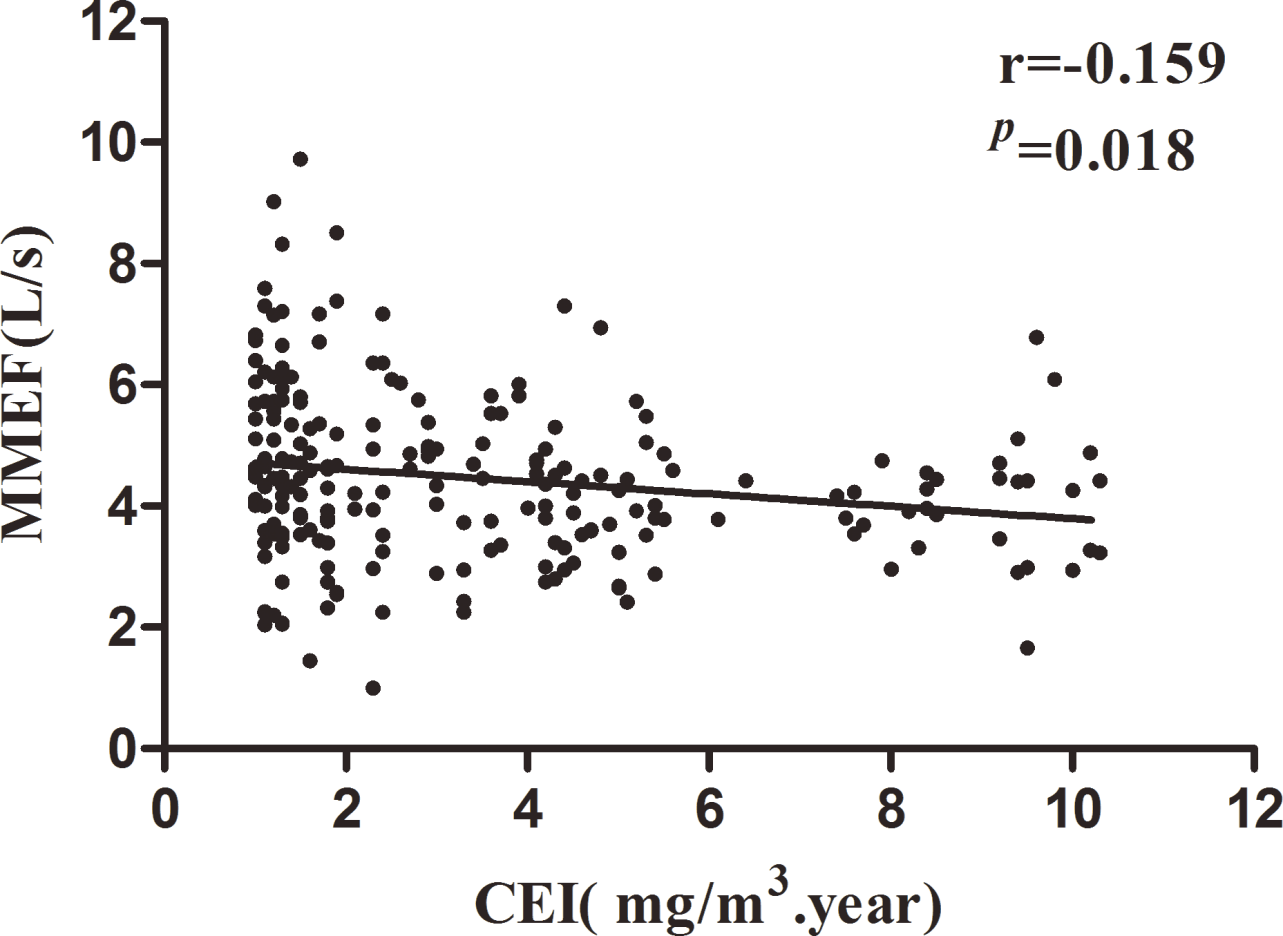
Correlation of maximal med-expiratory flow curve (MMEF) and the manganese cumulative exposure index (Mn-CEI) in male workers among high exposure group. Data were analyzed by partial correlation, r = -0.159, *p*<0.01.

In the correlation analysis, a decreased linear trend of MMEF was found in the exposed subjects with the increased dose of Mn-CEI in the scatter diagram, and a significant negative correlation between MMEF and CEI (r = -0.159, *p*<0.05) was shown among smoking workers in high exposure group, as shown in Fig. 1.

## Discussion

In order to explore potential adverse effects of manganese dust exposure on lung function in ferromanganese works, we investigated 1658 workers who were employed in a ferromanganese refinery in Guangxi. We also addressed potential interaction between smoking and manganese dust exposure, whether smoking would modify the impact of manganese dust exposure on lung functions.

Soyseth et al. and Antonini et al. found that manganese dust exposure adversely affects pulmonary function including airflow limitation, bronchitis, airway irritation, lung function changes [23,24]. Our data also found that ferroalloy working was related to reduce lung ventilation function. Our main finding was that long-term Mn dust exposure for male workers was related to a significant decrease in FEV_1_, FEV_1_% pred, MMEF and MMEF% pred, FEV_1_/FVC, MVV and MVV% pred. FEV_1_ is the main indicator of pulmonary function damage and FEV_1_/FVC is a parameter of obstructive pulmonary dysfunction, These results might indicate potential impairment of medium and large airways, and an increase in the prevalence of obstructive ventilator disorder among male workers [25]. Loukzadeh et al. [26] and Bowler et al. [22] also found that the average of FEV_1_ and FEV_1_/FVC were significantly lower in high exposure welders than low exposure welders. However, Johnsen [27] found that only FEV_1_ annual declined among employees in the smelting industry; the main reason may be differences in lung function measurement methods and basic characteristics of the study population. In addition, we did not find Mn dust exposure adversely affected pulmonary function among female workers. This may be due to women generally being assigned to tasks that had lower manganese exposure [28] and possible stronger healthy worker effects among women [29]. In this cohort population, we explored the potential synergistic effect between smoking and Mn dust exposure but the results showed that lung function was only statistically significant reduced in FVC% pred, MMEF and MMEF% pred in the higher exposure group. This is in consistent with findings of studies accomplished by Boojar et al.[4] and Beckett et al. [30] which effects was only seen in exposed workers compared with controls. Like our study, Sharifian et al. found no significant decreased pulmonary function parameters (FEV_1_, FEV_1_/FVC, PEFR) and smoking [31]. The lung function of smoking and non-smoking workers was similar in the low exposure group. These results, however, might be interpreted with caution because the low exposure group population was younger than 40 years, and exposure duration was probably not long enough for airflow limitation to become apparent [32]. Nicola et al. found that smoking history was not associated with pulmonary function decline in young smokers, which might be due to the pulmonary function tests being unable to detect early physiologic changes in the airways [33].

The MMEF is particularly reduced when the small airways function is impaired, as well as parenchymal damage [34]. In the correlation analysis, a significant correlation between MMEF, MMEF% pred and Mn-CEI was shown among smoking workers in the high exposure group. These results might indicate the function of small airways was injured due to an increase in mucosal secretions and perhaps decrease in the diameter of respiratory ducts. Similarly, Nemoto et al. [35] epidemiologically demonstrated the harmful effect of long-term cigarette smoke inhalation on MEFs in male smokers. Dorman et al. [7] also reported that high-dose subchronic manganese sulfate inhalation was associated with small airway inflammatory changes in the absence of observable clinical signs. In addition, this study did not find a negative correlation between cumulative exposure to Mn and FVC, FEV_1_, but the lung function of male workers has changed. Therefore, we need to continually follow up this aspect among regarding workers.

Our study had some limitations. First, this survey was designed as a retrospective study, therefore, further prospective studies are needed to better clarify the nature of the observed association and even other lung diseases such as asthma and COPD. Second, we only evaluated the effects of manganese dust on lung function of workers, and we did not collect other potential harmful factors which affected lung function, such as harmful gas including carbon monoxide and nitrogen oxide.

## Conclusions

This study showed that the risk of decreasing lung function was associated with occupational cumulative exposure to manganese dust in male smelter workers, and individual smoking habits and manganese exposure had a synergistic effect on the lung function decrease. No significant relationship was found between female workers and manganese dust exposure. In order to investigate the relationship between occupational exposure and impairment of lung function, longitudinal studies are required in our cohort.

## Supporting Information

S1 Table Manganese dust concentration PC-TWA in the different types of work of the GXMEWHC. (DOC)

S2 Table The Mn-CEI of the Guangxi manganese-exposed workers healthy cohort (GXMEWHC). (DOC)
