# Supplementary material for: Synergistic Impaired Effect between Smoking and Manganese Dust Exposure on Pulmonary Ventilation Function in Guangxi Manganese-Exposed Workers Healthy Cohort (GXMEWHC)
Source: PLoS One. 2015 Feb 9;10(2):e0116558. doi: 10.1371/journal.pone.0116558 (PMC4321994; doi:10.1371/journal.pone.0116558)
Supplement: S1 Table — (DOC) [file pone.0116558.s001.doc]

**S1 Table Manganese dust concentration PC-TWA in the different types of work of the GXMEWHC**

|  |  |  | **TWA（mg/m^3^ ）** | |
| --- | --- | --- | --- | --- |
| **Types of work** | **Number (n)** | **Per cent (%)** | **Median (IQR)** | **Range** |
| Smelter | 477 | 28.8 | 0.165(0.083) | 0.015 –0.363 |
| Human crushing worker | 269 | 16.2 | 0.308(0.001) | 0.240 –0.309 |
| Craneman | 70 | 4.2 | 0.210(0.323) | 0.027 –0.352 |
| Finishing machining worker | 84 | 5.1 | 0.082(0.003) | 0.082 –0.140 |
| Scaleman | 99 | 6.0 | 0.108(0.069) | 0.062 –0.177 |
| Sampleman | 19 | 1.2 | 0.042(0.001) | 0.040 –0.042 |
| Welder | 111 | 6.7 | 0.068(0.001) | 0.067–0.068 |
| Chemical analyst | 52 | 3.1 | 0.068(0.020) | 0.068 –0.088 |
| Repairman | 148 | 8.9 | 0.021(0.002) | 0.021–0.022 |
| Electrician | 88 | 5.3 | 0.016(0.025) | 0.004 –0.041 |
| Alkali recovery worker | 128 | 7.7 | 0.027(0.003) | 0.024 –0.045 |
| Car driver | 113 | 6.8 | 0.024(0.001) | 0.027 –0.030 |
| Total | 1658 | 100 | 0.088(0.221) | 0.004 –0.363 |
| PC-TWA, permissible concentration-time-weighted average. | | | | |
