# Supplementary material for: Synergistic Impaired Effect between Smoking and Manganese Dust Exposure on Pulmonary Ventilation Function in Guangxi Manganese-Exposed Workers Healthy Cohort (GXMEWHC)
Source: PLoS One. 2015 Feb 9;10(2):e0116558. doi: 10.1371/journal.pone.0116558 (PMC4321994; doi:10.1371/journal.pone.0116558)
Supplement: S2 Table — (DOC) [file pone.0116558.s002.doc]

**S2 Table The Mn-CEI of the Guangxi manganese-exposed workers healthy cohort (GXMEWHC)**

| **Mn-CEI (mg/m^3^ year)** | **Number (n)** | **Per cent (%)** | **Median (IQR)** | **Range** |
| --- | --- | --- | --- | --- |
| Low exposure group (0<Mn-CEI<1) | 682 | 41.1 | 0.417 (0.486) | 0.010 –0.990 |
| High exposure group (Mn-CEI>1) | 976 | 58.9 | 2.513 (2.108) | 1.000 –10.300 |
| Total | 1658 | 100 | 1.290 (2.291) | 0.010 –10.300 |

Mn-CEI, cumulative exposure index.
